# Malaria prevention practice and associated factors among residents in Fogera district, Ethiopia, during the armed conflict between Amhara Fano forces and the federal government, 2025

**DOI:** 10.1186/s12936-026-06035-3

**Published:** 2026-07-21

**Authors:** Masresha Kassaw Ewinetu, Bekalu Mekonen Belay, Yirgalem Abere

**Affiliations:** Department of Adult Health Nursing, College of Health Science, Debre Tabor University, Debre Tabor, Ethiopia

**Keywords:** Malaria, Practice, Prevention and control, Bed net, Utilization

## Abstract

**Background:**

Malaria is continuously a great health challenge in Ethiopia, which is worst during armed conflict. Hence, continuous malaria prevention practice is very essential. However, there is no evidence of malaria prevention practices among the residents in Ethiopia during armed conflict. Thus, this study aimed to provide tangible evidence regarding malaria prevention practices and associated factors among residents in Fogera district, Ethiopia, during the armed conflict between Amhara Fano and the federal government, 2025.

**Method:**

A community-based cross-sectional study was conducted among residents in Fogera district from September 1–30, 2025. Multistage sampling was applied to select households. The data was collected by trained nurses via face-to-face interview and analyzed using SPSS version 25. Data were summarized with frequency, percentage, mean and standard deviation for both continuous and categorical variables. All predictors with a p-value of less than 0.25 in bi-variable analysis were entered in to multivariable logistic regression to control the confounding effect. An AOR with 95% CI and a p-value of < 0.05 was used to announce statistically significant.

**Result:**

A total of 804 participants were included with a 99.5% response rate. 48% of respondents had good practice of malaria prevention and control interventions. Predictors including male gender (AOR = 1.41, 95% CI 1.10–1.81), urban residence (AOR = 1.50, 95% CI 1.01–5.92), having bed net (AOR = 6.91, 95% CI 3.79–8.59) and adequate knowledge (AOR = 2.32, 95% CI 1.34–4.03) were significantly associated with malaria prevention practice.

**Conclusion:**

Almost half of the residents had poor practice of malaria prevention and control methods and one-third of the participants had no bed net. Hence, health officials and partners need to give more attention by providing ongoing supervision, support and access to a bed net.

## Introduction

Malaria is a vector-borne disease caused by protozoan parasites transmitted through the bites of infected female Anopheles mosquitoes [1]. Malaria can affect all individuals who have contact with mosquitoes. However, it is very dangerous to pregnant women, children and immunosuppressive patients. Hence, vector control and case management are crucial [2].

Malaria remains a major public health challenge in Ethiopia, particularly during periods of armed conflict [3]. Effective prevention practices, including the consistent use of insecticide-treated nets (ITNs), indoor residual spraying, environmental sanitation, and timely healthcare-seeking behavior, are essential for reducing malaria morbidity and mortality [4]. However, the effectiveness of these interventions largely depends on community participation and adherence to recommended preventive measures. In conflict-affected settings, disruptions to health services, population displacement, reduced access to preventive resources, and insecurity may adversely affect malaria prevention efforts [5]. Therefore, this study aimed to assess malaria prevention practices and identify associated factors among residents of Fogera District, Ethiopia, during the armed conflict between the Amhara Fano forces and the federal government.

Malaria prevention practices are influenced by a complex interaction of socio-demographic, economic, behavioral, environmental, and health-system-related factors [6]. Previous studies conducted in Ethiopia have identified factors such as educational status, knowledge about malaria, attitudes toward prevention methods, access to health information, and availability of preventive tools as significant determinants of malaria prevention behavior [7]. In conflict settings, additional challenges such as population displacement, interruption of healthcare services, insecurity, and limited access to malaria prevention resources may further affect community practices [5]. Understanding the factors associated with malaria prevention practices among residents of Fogera District during the armed conflict is essential for identifying vulnerable groups and designing targeted interventions that can strengthen malaria prevention and control efforts during humanitarian emergencies and periods of instability. Malaria prevention behavior in Ethiopia has been shown to be influenced by knowledge, attitudes, education, and access to preventive tools, and service disruptions, factors that may become even more critical during conflict situations [8].

In 2019, 229 million malaria cases and 409,000 malaria-related deaths occurred worldwide. Of those, 94% was contributed by Africans [9]. Similarly, it has become a big health challenge in Ethiopia. In 2024, the Ethiopian federal ministry of health reported a rise in malaria cases, 7.3 million cases and 1100 deaths, which is above reported in 2023 [10]. This is due to above 75% of the country's land mass is malarious, which makes the population more at risk of malaria [11]. Continuous practice of malaria prevention methods is crucial.

Malaria has a great impact on the health and prosperity of countries, communities, families and individuals [12]. It increases patient visits, health facility admissions, and the death rate and affects the socio-economic growth of society [12]. Now, malaria is identified as a cause and disease of poverty worldwide. According to economists, countries that have a high rate of malaria transmission can reduce their economic growth by 1.3% per person per year [13].

The Ethiopian ministry of health has set goals to eliminate malaria by 2030 and major progress has been made in decreasing malaria-related suffering by scaling-up malaria prevention strategies like insecticide-treated nets and indoor residual spraying of households, scale-up testing tools, environmental management, and updated treatment approach [14]. However, in Amhara regional state specifically the study area, malaria cases have increased [15]. On the other hand, malaria prevention and control practice is becoming shrinking related to armed conflict [15].

Conflict is the worst event that creates a complex situation for society, increasing disease outbreaks like malaria due to preventive practice undermining [16]. Armed conflict disrupts the health delivery system, distracts the infrastructure, incapacity disease surveillance and restricts access to important prevention methods. Through war, residents' shelters are damaged that to migrate to short-term shelters that lack enough protection against mosquito exposure [17]. Even if they are accessed, their utilization is inconsistent or ineffective.

In addition to this, the war adversely affects individuals' understanding, view, and practices of malaria prevention and control measures. Health education activities are often suspended, and the community lacks information about malaria prevention and control. The community is exposed to economic deprivation, food insecurity and stress that contribute to poor adherence to recommended malaria prevention practices. This is worse for vulnerable groups like women, children and migrant societies who were affected unfairly, and they account for the major morbidity and mortality related to malaria [18]. Hence, knowing of malaria prevention and control practice is very essential for establishing suitable intervention methods during war to safeguard those vulnerable societies and to achieve malaria prevention goals.

Even though there is clearly recorded evidence that conflict affects the practice of malaria prevention and control, there is no evidence that shows malaria prevention and control practice in Ethiopia, specifically the study area. Therefore, this study aimed to investigate malaria prevention practice and associated factors among residents in Fogera district, Ethiopia, during the armed conflict between Amhara Fano and the federal government, 2025.

## Methods and materials

### Study setting, design and period

A community-based cross-sectional study was conducted in Fogera district, Amhara regional state, Ethiopia, from September 1–30, 2025. Fogera district is one woreda under the South Gondar zone, which is found 625 km north of far from Addis Ababa, Ethiopia. Fogera is flat and low land which the altitude of the district ranged between 1750 and 2100 m above sea level. The district gains a major portion of rainfall between June and October [15].

The district has 30 rural and 5 urban kebeles (meaning smallest administrative unit). Based on the housing and population census, the projected population of Fogera district in 2022 was estimated to be 300,473 people and 52,905 households [15]. Almost all parts of the district are malarious and affect the population throughout the year. In the district, there is one primary hospital, eight health centers, and thirty-eight health posts.

### Study population, sample size and sampling technique

The source population for this study was all households live in Fogera district. On the other hand, the study population was all arbitrarily selected households living in the selected kebeles. Adults who were 18 years and above, who were resident in the kebele for at least 6 months and who gave consent were included. Whereas, all adults who did not give consent and were critically ill during the data collection period were excluded from the study.

Sample size was calculated for the first objective through a single population proportion formula by considering these assumptions: proportion (51%) which had been taken in previous studies in Andasa district [19], 95% CI and 5% tolerable error. For the second objective, three sample size was calculated with Epi-info 7.2 software by considering major associated factors from previous literature, such as sex, income level and occupation [19]. The largest sample size with 6% non-response was used and multiplied by 2 for design effect; the final sample size became 814.The sample was allocated proportionally for the six randomly selected kebeles and systematic random sampling technique was employed in order to select individual households from each kebeles.

### Data collection tools, procedures and quality assurance

In the very beginning, a structured and pretested questionnaire was adapted from previous literature [7, 20, 21]. The questionnaire assessed socio-demographic (9), knowledge (10), attitude (10) and malaria prevention practice (10). Practice was assessed by three likert scale questions which had three possible levels ranging answers: always (score 3), sometimes (score 2) and never (score 1).

Primarily, the questionnaire was prepared in the English version, then translated to the local language ‘Amharic’, then retranslated back to the English version to evaluate the consistency of the data. Two days' training was given to six diploma-holder nurses for data collectors and two B.Sc. degree holder Nurses to supervisor about the method of data collection with interview and how to fill the information that is given by the participants.

A pre-test was done in Adiszemen district for 10% of the sample size, and then verbal informed consent was obtained from each participant. Finally, the actual data was collected with an Amharic version questionnaire by interviewing the head of the household. If not availed, a responsible adult who is 18-years or older was interviewed.

### Study variables and operational definition

The dependent variable was the practice of malaria prevention and the predictors include age, sex, marital status, educational level, family size, occupational status, residence, income level, knowledge and attitude towards malaria prevention practice.

Malaria prevention practice: Respondents who scored ≥ 50% of the right answers in the total malaria prevention practice assessment questions were categorized as having good practice. Whereas, respondents who scored < 50% of practice assessment questions were categorized as poor practice [7].

### Data processing and analysis

Finally, sufficiency and uniformity of the data was examined. Next to this, the data guide format was developed, entered into Epi-data entry 3.1 software and exported to SPSS version 25 for analysis. Data were summarized with frequency, percentage, mean and standard deviation for both continuous and categorical variables. Bivariable and multivariable logistical regression was applied to recognize the feasible predictors that influence the practice of malaria prevention methods. All predictors with a p-value of less than 0.25 in bi-variable analysis were entered into multivariable logistic regression for extra scanning to control confounding effect. An AOR (meaning adjusted odds ratio) with 95% confidence intervals and a p-value of < 0.05 was used to announce statistically significant.

## Result

### Socio-demographic data

In this research, as a whole, 804 households engaged with a response rate of 99.5%. The mean age of the participants was 31.11 (SD ± 11.2) years, and ranged from 18 to 75 years. Among the participants, 550 (68.4%) were male and 513 (63.80) lived in rural parts of the district. The greater proportion of the study participants, 682 (85%), were orthodox Christians, and the vast majority of participants, 616 (76.61%) were wedded. Regarding the educational level, most of the participants, 62.68% do not able to write and read (Table 1).

**Table 1:** Socio-demographic characteristics of residents in the selected households from Fogera district, Ethiopia, 2025 (n = 804)

Characteristics	Category	Frequency	Percent
Age (year)	18–28	326	40.50
	29–39	354	44.00
	40–50	81	10.07
	≥ 51	43	5.35
Sex	Male	550	68.4
	Female	254	31.6
Marital status	Married	616	76.61
	Single	86	10.69
	Divorced	63	7.83.6
	Widowed	39	4.85
Religion	Orthodox	682	84.82
	Muslim	79	9.82
	Protestant	23	2.86
	Catholic	20	2.48
Level of education	No formal education	504	62.68
	Primary (1–8 grade)	203	25.24
	Secondary (9–12 grade)	60	7.46
	College and above	37	4.6
Occupation	Employee	30	37.31
	Merchant	153	19.02
	Farmer	563	70.02
	House wife	58	7.21
Family members	1–3	410	50.99
	4–6	280	34.82
	≥ 7	114	14.18
Monthly income in birr	≤ 2000	511	63.55
	2001–4000	210	26.11
	≥ 5001	83	10.32
Residence	Rural	513	63.80
	Urban	291	36.19

### Knowledge about malaria prevention methods

Among all study participants, nearly two-thirds (59.56%) of participants had an adequate level of knowledge about malaria prevention and control methods. Almost three-fourths of participants, 612 (76.11), and 600 (74.62%) understood that they can prevent malaria transmission through drainage of stagnant water and use of bed nets. On the other hand, half of the participants understood that malaria is transmitted from person to person (Table 2).

**Table 2:** Knowledge of the study participants towards malaria prevention and control methods in Fogera district, Ethiopia, 2025 (n = 804)

Variables	Yes		No	
	No	%	No	%
Fever, joint pain, headache are the symptoms of malaria	603	75.00	201	25.00
Malaria is transmitted from person to person	400	49.77	404	50.23
Malaria is transmitted through mosquito bite?	613	76.24	191	23.75
Malaria breeds in stagnant water and discarded materials	400	49.75	404	50.25
Mosquitoes mostly bite persons at night	504	62.68	300	37.31
Anti-mosquito spray in the house can prevented malaria breed	325	40.42	479	59.57
Use of bed net can prevent malaria totally	600	74.62	204	25.37
Drainage of stagnant water can reduce malaria transmission	612	76.11	192	23.88
Clean the environment can reduce malaria transmission	490	60.94	314	39.05
Pregnant mothers and children are more risky to malaria	329	40.92	475	59.07
Knowledge	Adequate		479 (59.56%)	
	Inadequate		325 (40.44%)	

### Attitude towards malaria prevention and control among households

Arising out of the entire study participants, 58% (95% CI 53.8–61.8) of study members expressed positive attitudes to malaria prevention and control practice. Three-thirds of the study members, 633 (78.71%) and 702 (87.3%), trust that every individual is at risk for malaria and can prevent it through mosquito bed nets, respectively. On the contrary, two-thirds of the participants, 501 (62%) believe malaria is not an avoidable disease (Table 3).

**Table 3:** Knowledge of Self-Care Behavior among Adult Cancer Patients Receiving Chemotherapy Treatment at the Oncology Unit in Amhara Region, Ethiopia, 2024 (n = 403)

Variables	Yes	No
	No (%)	No (%)
Consulting health care provider during vomiting	339 (84.1)	64 (15.9)
Skin rash or lesion cannot be treated by scratching the lesion.	269 (66.7)	134 (33.3)
Brushing the teeth after every meal is necessary.	336 (83.4)	67 (16.6)
Not taking food or fluid immediately before and after chemotherapy.	221 (54.8)	182 (45.2)
Brushing the tooth with soft toothbrush is advisable	342 (84.9)	61 (15.1)
Breathe deeply and slowly when you feel nauseated.	241 (59.8)	162 (40.2)
Dry mouth and lips can be treated by drinking plenty of water	337 (83.6)	66 (16.4)
Avoid using cosmetics & sprays during chemotherapy treatment.	268966.5)	135 (33.5)
Wear thick socks & soft sole shoes can prevent tingling of the toe	342 (84.9)	61 (15.1)
Fatigue can be prevented or treated by taking rest or snap	257 (63.8)	146 (36.2)
Wash hands before and after meals can prevent infection	319 (79.2)	84 (20.8)
Not recommended to participate in community meetings	234 (58.1)	169 (41.9)
Avoid lying down immediately after eating can prevent nausea	322 (79.9)	81 (20.1)
Not taking long hot baths can prevent tingling of fingers and toe	210 (52.1)	193 (47.9)
Eating sour food like lemon can improve appetite.	321 (79.7)	82 (20.3)

### Practice of malaria prevention and control among households

The practice of malaria prevention and control among residents was categorized into good and poor levels. From the total of eight hundred-four participants, only 386 (48%) had good malaria preventive and control practices, with a 95% confidence interval of 43.2–52.9. As we have seen from the participants' daily malaria prevention practice, only 109 (13.55%) used bed net daily, the rest 205 (25.49%) of the participants use bed net sometimes which might not be totally prevent malaria transmission. About two-thirds of the participants had never used a net at night, even though they had a bed net. And also, about seventy percent of the study participants never used a strength indoor residual spraying agent. Among the participants, 615 (76%) always visit a health institution when they feel sick; the rest 11% buy drugs from drug stores (Table 4).

**Table 4:** Malaria prevention practice among residents in Fogera district, Ethiopia, 2025 (n = 804)

Item	Never	Sometimes	Every time
	N (%)	N (%)	N (%)
How often do you sleep in a mosquito bed net?	490 (60.94)	205 (25.49)	109 (13.55)
How often do other members of the household sleep in bed nets?	481 (59.82)	245 (30.47)	78 (9.7)
How often do you check for holes/repair mosquito nets?	645 (80.22)	130(16.16)	29 (3.6)
How often do you use mosquito-repellent coils in your house?	643 (79.97)	150 (18.65)	11 (1.3)
How often do you use an anti-mosquito spray in your house?	585 (72.76)	210 (26.11)	9 (1.11)
How often do you clean/cut bushes around your house?	102(12.68)	565 (70.27)	137 (17.0)
How often do you drain stagnant water near your house?	212 (26.36)	535 (66.54)	57 (7.9)
How often do you wear clothes that cover all your body at night?	28 (3.50)	95 911.81)	681 (84.7)
How often do you close your door at 12 o’clock?	105 (13.06)	514 (63.93)	185 (23.3)
How often do you go to health institution when experience fever?	85 (10.57)	104 (12.93)	615 (76.4)

### Factors associated with good practice of malaria prevention and control

After managing the possible confounding, determined by multivariable logistic regression analysis, four variables, including male gender (AOR = 1.41, 95% CI 1.10–1.81), urban residence (AOR = 1.50, 95% CI 1.01–5.92), having bed net (AOR = 6.91, 95% CI 3.79–8.59), and adequate knowledge (AOR = 2.32, 95% CI 1.34–4.03) were importantly associated with malaria prevention practices.

Accordingly, male participants were almost two times more likely to have good malaria prevention practice as compared to females (AOR = 1.41, 95% CI 1.10–1.81). Participants who were urban residence had one pint five times more likely to have good malaria prevention practice than rural residences (AOR = 1.41, 95% CI 1.10–1.81). The odds of good malaria prevention practice were nearly seven times higher among participants who had a bed net as compared to those who did not have a bed (AOR = 6.91, 95% CI 3.79–8.59). Likewise, participants who had a good level of knowledge about malaria prevention methods were nearly two times more likely to have good malaria prevention practice than those have poor knowledge about malaria prevention methods (AOR = 2.32, 95% CI 1.34–4.03) (Table 5).

**Table 5:** Multivariable logistic regression analysis for malaria prevention practice and associated factors among residents in Fogera district, Ethiopia, 2025 (n = 804)

Variables	Malaria preventive practice		AOR (95% CI)	p-value
	Good N (%)	Poor N (%)		
Age				
18–28 years	182 (22.63)	112 (13.93)	3.60 (0.58, 8.23)	0.07
29–39 years	140 (17.41)	170 (21.14)	1.73 (0.40,5.30)	0.06
40–50 years	74 (9.20)	70 (8.70)	2.1 (0.72–3.4)	0.21
Above 50	17 (2.11)	39 (4.85)	1	
Sex				
Male	242 (35.2)	308 (27.8)	1.41 (1.10,1.81)	0.000**
Female	80 (12.9)	174 (24)	1	
Residence				
Urban	105 (13.05)	186 (23.13)	1.50 (1.01,5.92)	0.000**
Rural	153 (19.02)	400 (49.75)	1	
ITN ownership				
Yes	320 (39.80)	280 (34.82)	6.91 (3.79,8.59)	0.001**
No	30 (7.44)	174 (21.64)	1	
Knowledge				
Adequate	347 (43.15)	132 (16.41)	2.32 (1.34,4.03)	0.003**
Inadequate	160 (19.90)	165 (20.52)	1	
Attitude				
Favorable	334 (41.54)	138 (17.16)	2.19 (0.34,3.48)	0.067
Unfavorable	163 (20.27)	169 (21.01)	1	

The symbol (**) indicates variables that are statistically significant

## Discussion

In this study, the overall good malaria preventive practice among residents of Fogera district during the law enforcement operation period was 48%, which is less than fifty percent. This is comparable with findings that have been documented in Andasa cluster, Ethiopia, Jawi district, Ethiopia, Hawasa City, Ethiopia and Ghana [19, 22–24]. This is due to; In Africa there is a similarity of socioeconomic status and malaria preventive strategies. However, the results of this study are greater compared with the previous findings from Ghana and Senegal [25, 26]. On the contradiction, this finding is lower than findings in Arba Minch, Ethiopia, Nigeria, Cameron and Myanamar [24, 27–29]. This discrepancy might be due to variation in socio-economic, health service and study population.

This study detected that less than half, 39%, of study participants are sleeping underneath an ITN. Of those, pregnant people and children use the greater percentage of bed utilization, which is 70%. This is lower than the target of the national operational plan for malaria, which was planned to have levels of above 80%. This could be due to a scarcity of ITNs at the household level and armed conflict disrupts the health delivery system of the country, and restricts access to important prevention instruments. This is lower than findings in Chagni, Ethiopia and Zambezia [30, 31]. However, these findings are higher than the findings in Senegal and Uganda [26, 32]. This variation might be due to variation in the local malaria burden, dominant mosquito species and their behaviors, variation in local climate and infrastructure for health care.

In this study, male participants were 1.4 times more likely to have good malaria prevention practice in comparison to equivalents. This is similar to discoveries from the investigation in Belesa, Ethiopia [33]. This variation is due to social, economic and cultural features but not due to biological variation. Men frequently have significant access to health information through community meetings, the media and the workplace, which can increase their awareness of prevention methods and practice. Also, in many communities, men guide household resources which allow them to buy preventive tools like bed nets and house spray that, over all, increase malaria preventive practice. On the other hand, women have a heavier household workload, give priority to children to sleep under bed nets, and face restrictions on participating in health initiatives like practicing of preventive measures.

The study members from urban resident were almost 1.5 times more likely to have good practice of malaria prevention as compared to rural residents. This is similar with findings from a study in Hawasa Ethiopia, Southern Tanzania, Cameroon and Malawi [7, 34–36]. The reason might be that urban dwellers have better access to health information and health service since they usually live closer to health institutions. Thus, they have exposure to mass media (TV, radio, social media) that improves awareness and level of malaria prevention practice. In addition to this, they have access to bed nets, indoor residual spraying, and health education on how they prevent malaria transmission. The other reason, urban residents have some how much improved house condition (screened windows and doors, closed eaves, cement walls and roofs) and better environmental management (organized waste disposal, drainage system) that might be increase individuals malaria prevention practice.

In our study, participants who had adequate knowledge about malaria prevention were 2.3 times more likely to have good malaria prevention practices than their counterparts. This is in line with findings from the study in Hawasa, Ethiopia and Malawi [7, 36]. This is because understanding directly impacts on individuals' views, commitments and habits. Knowledgeable individuals can identify the seriousness of malaria, risk groups, effective prevention methods and build confidence in the effectiveness of the preventive measures that end up with increased practice of preventive actions. And also, individuals who are well-informed about malaria control methods do not believe misconceptions which can lead to proper preventive practices.

Participants who have a bed net were almost 6.9 times more likely to have good malaria prevention practice as compared to their counterparts. This finding is similar to the findings from the study in Wolita, Ethiopia, Gurage zone [37, 38]. This dissimilarity is because having a bed net always comes after health education about malaria transmission and prevention, which inspires them to practice malaria preventive measures additionally. In addition to this, an own bad net indicates that an individual is earlier health aware. Those individuals avoid mosquito breeding sites, more agreeable indoor residual spraying and use repellents or protective clothing.

## Conclusion

The overall malaria prevention practice among households is 48% which is insufficient as compared with other national findings and the pre-decided Ethiopian operational plan for malaria prevention. Only thirty-nine percent of the households were reported that they prevent malaria through the use of ITN, while thirty-seven percent through anti-mosquito spray. Being male, an urban resident, having a bed net and a good level of knowledge of malaria prevention and control were positively associated with malaria prevention practices.

Therefore, further to increase the practice of malaria prevention measures undertaken at the house hold level, ITN distribution programs should be strengthened, the house to house spray program should cover larger geographical areas and the community should take actively participate in community campaign programs. Furthermore increase academic knowledge should be considered to address those who have lack of information and who are illiterates. Further, direct observational study is better to address the bottle neck of this research.

### Strength and limitations of the study

Being a community-based study and using a large sample size enhance the generalizability of the study findings. However, cross- sectional design of the study can lead to some bias including recall bias, and reflects information collected at a specific time point, and this information may be different in another period. However, the study has been conducted during the malaria transmission season to minimize this recall bias. In addition, the estimates could have been underestimated or overestimated due to the fact that certain information collected in the study was self-reported by the participants. For instance, the use of bed nets was self-reported and could have been overestimated by the participants for social desirability. It is shown that self-reports overestimate the use of bed net.

## Data Availability

All data relevant to the study are included in the article.
